# Increased Cell Fusion in Cerebral Cortex May Contribute to Poststroke Regeneration

**DOI:** 10.1155/2013/869327

**Published:** 2013-04-03

**Authors:** Alexander Paltsyn, Svetlana Komissarova, Ivan Dubrovin, Aslan Kubatiev

**Affiliations:** ^1^Institute of General Pathology and Pathophysiology of the Russian Academy of Medical Sciences, Baltiskaya Street 8, Moscow 125315, Russia; ^2^Russian Medical Academy of Postgraduate Education, Moscow, Russia

## Abstract

In this study, we used a model of a hemorrhagic stroke in a motor zone of the cortex in rats at the age of 3 months The report shows that cortical neurons can fuse with oligodendrocytes. In formed binuclear cells, the nucleus of an oligodendrocyte undergoes neuron specific reprogramming. It can be confirmed by changes in chromatin structure and in size of the second nucleus, by expression of specific neuronal markers and increasing total transcription rate. The nucleus of an oligodendrocyte likely transforms into a second neuronal nucleus. The number of binuclear neurons was validated with quantitative analysis. Fusion of neurons with oligodendrocytes might be a regenerative process in general and specifically following a stroke. The appearance of additional neuronal nuclei increases the functional outcome of the population of neurons. Participation of a certain number of binuclear cells in neuronal function might compensate for a functional deficit that arises from the death of a subset of neurons. After a stroke, the number of binuclear neurons increased in cortex around the lesion zone. In this case, the rate of recovery of stroke-damaged locomotor behavior also increased, which indicates the regenerative role of fusion.

## 1. Introduction

Protection, rehabilitation, and stroke outcome are determined by the extent of the preserved neuronal tissue. Thus, the maintenance and regeneration of stroke-injured neurons is a prominent topic on which there are many publications, all of which represent neuronal regeneration exclusively as a result of neurogenesis. This tendency can be justified only in one case, when a stroke occurs in the dentate gyrus (fascia dentata hippocampus) or in the olfactory bulb. These two zones are reasonably considered neurogenic because they are sites of the replacement of granular neurons. Granular neurons are formed in two other neurogenic zones: the subgranular layer of the dentate gyrus [1–5] and the subventricular layer of the cerebral ventricles [6–8]. Neuroblasts migrate from these zones to the granular layer of the dentate gyrus [9–11] and to the olfactory bulbs [8, 12], where they differentiate into granular neurons. Reports of neurogenesis in other brain regions, as in the review of Gould [13], contradict other experiments [14–17]. Therefore, scientific consensus purports that, in other brain regions, neurogenesis does not occur. According to one hypothesis, neurogenesis does not normally occur in the cortex but appears after stroke [18, 19]. However, some publications do not confirm this point of view [20]. These issues diminish the impact of neurogenesis research and stimulate studies of well-quantified regenerative mechanisms after cortical stroke. Only studies of the true, fundamental, clearly detectable mechanisms of brain regeneration can become a basis for the development of effective therapeutic strategies. In our previous research [21–23], we described the phenomenon of the fusing of neurons with oligodendrocytes. We found an increase in the number of fusing instances in a zone with ischemic damage on a method Watson et al. [24] of the cortex in rats. 

In the present study, we used the rate of formation of binuclear cells as a measure of neuronal regeneration. We demonstrated that, after fusion, a cell that contains two different nuclei (heterokaryon) is formed. The oligodendrocytic nucleus in the heterokaryon is subjected to neuron-specific reprogramming. The chromatin's structure and an expression of neuronal markers specify that the oligodendrocytic nucleus in the heterokaryon transforms into the neuron's second nucleus. We hypothesized that binuclear, structurally doubled neurons can perform double functions. The formation of binuclear neurons can compensate for the functional deficit caused by the death of a subset of neurons during stroke. In other words, the content of binuclear neurons, which can be easily counted, is a measure of regeneration in damaged cortex. Regeneration is stimulated by an injury. Here, we demonstrate an increase in the content of binuclear cells in the boundary and adjacent to lesion zones of the cortex.

## 2. Materials and Methods

### 2.1. Animals and Hemorrhagic Stroke Model

Experiments on male Wistar rats weighing 280–310 g were performed according to existing standards (National Standard of Russian Federation GOST P 53434-2009). The animals were subjected to a hemorrhagic stroke model that allowed for analysis of the functional changes that were caused by stroke [25]. To implement this model, the animals were anesthetized with ether, and the skin was dissected along the midline of the head. The periosteum was removed from the surgical area. A damaging object—a transplant—was inserted into the left motor cortex area (S1FL), which was responsible for right forelimb movements. The exact coordinates of the injury were determined according to a stereotaxic atlas [26]: 2 mm from the bregma in the rostral direction, 4 mm laterally from the cranium midline, and at a depth of 2.5 mm. The craniotomy was drilled with a bur, and the transplant was inserted by a catheter needle with a depth controller. The needle diameter was 1.7 mm, which was equal to the bur diameter. The needle was kept in the foramen for 3-4 minutes after the injection to prevent transplant efflux. Right forelimb paresis appeared in all of the operated on rats.

The transplant contained the following elements: homogenized hemostatic collagen sponge dispersed in phosphate-buffered saline (PBS) and Chinchilla rabbit with blood plasma free of cells (group 1) or platelet-rich rabbit plasma (PRP) (groups 2–5). The transplant volume was 300 *μ*L. In group 1, the transplant was injected as a 1 : 1 mixture of a collagen sponge and plasma. The platelet concentration was determined in PRP. Depending on the platelet content, the ratio of sponge to PRP volume was not strictly 50% but changed insignificantly to maintain a constant platelet quantity (6 × 10^7^) in the transplants for groups 2–5. The PRP was made of whole rabbit blood taken from the auricular vein using 9% sodium citrate (1/10 by volume) and centrifuged (Term BR4i with rotor S40) at 900 rpm for 10 minutes at 23°C. The supernatant, which contained PRP, was removed, and the pellet was centrifuged again at 2700 rpm for 10 minutes (23°C). After the second centrifugation, the supernatant containing cell-free plasma was obtained. 

### 2.2. Sample Preparation for Histology

#### 2.2.1. Material Fixing

Poststroke locomotion was tested two times: after the end of the operation and through 7 days. The brains were fixed in etherized rats after the second test of locomotor behavior. 

The catheter of the perfusion system was introduced into the left cardiac ventricle, and 15 mL of isotonic solution and 10 mL of 4% paraformaldehyde solution in PBS were perfused. The skull was dissected, and the brain was isolated and fixed with a 4% solution of paraformaldehyde in PBS. 

The locus of the transplant injection was visible in the isolated brain; therefore, the following manipulations were conducted in consideration of this point. 

#### 2.2.2. Vibratome Sections

Such sections allowed us to observe histologic changes in all of the areas of damage and in the surrounding intact cortex. Frontal 20 *μ*m sections were obtained using a vibratome (Leica VT 1000S, Austria) at the level of the cortical transplant injection. In the control group, frontal sections were cut from the S1FL field of the left hemisphere. The sections were placed on a microscope slide that was covered with poly-L-lysine and dried and stained using a 1% methylene blue ethanol solution for 10 minutes at room temperature. A further 10-minute staining was performed with mixtures of equal volumes of 0.25% cresyl violet solution and 0.5% toluidine blue solution. The stained sections were washed with distilled water and differentiated with 96% ethanol. 

#### 2.2.3. Location of Lesion and Perilesional Zones

**Figure 1:**
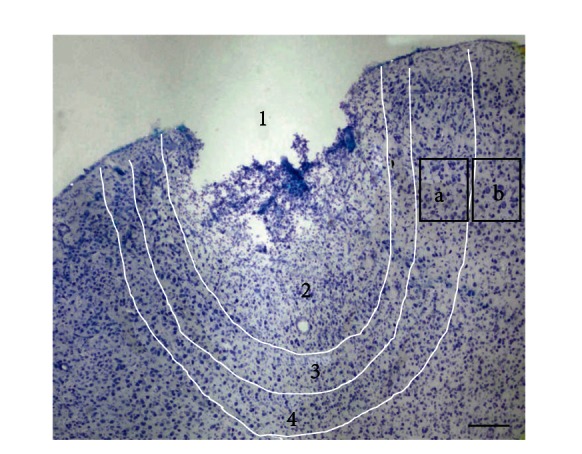
Location of lesion and perilesional zones of hemorrhagic stroke (S1FL field of cortex)—1: transplant (crumbled out), 2: infiltrate, 3: penumbra, 4: boundary zone. Rectangular areas of cortex from which tissue was taken for analysis—a: in the boundary zone; b: in the adjacent cortex. The scale bar is 300 *μ*m.

The histological treatment removed most of the transplant (sometimes together with a part of the infiltrate). The empty space of the former transplant was surrounded by an infiltrate zone. It contained only cells of the infiltrate and vessels; neurons were absent. More peripheral penumbra was observed. Here we found edema and cells of infiltrate and neurons, among which alive and dead cells were observed according to their morphology. A layer of cortex located outside the penumbra is the boundary zone. Here, it was possible to find only vascular changes compared with surrounding normal-morphology cortex.  Figure 1 shows a part of the cortex that had insult zones (Figure 1). 

Angiogenesis was identified by the following signs. In infiltrate can only be newly formed vessels. In penumbra vessels quantity surpassed their number in the intact cortex. On this fact we drew a conclusion about the quantity of these vessels that appeared in the angiogenesis.

#### 2.2.4. Semithin Sections

Samples measuring 1 mm^3^ were cut and embedded into epoxy resin to increase the accuracy of the morphological analysis in the infiltration zones, the penumbra, and the boundary zone. The samples were treated to obtain semithin (1 *μ*m) sections. For calculation of the number of fusions, samples of cortex were analyzed separately according to their location: (a) within the boundary zone and (b) 1 mm lateral (Figure 1). In these samples, we investigated neurons from layers III–V. Semi-thin sections were obtained using an ultramicrotome (Leica EM UC6, Austria). The semithin and vibratome sections were analyzed in a light microscope, Olympus BX51 (Olympus Japan), with a camera Color View II. For photography and image analysis, we used Imaging Software for Life Science Microscopy Cell F (Olympus Japan). 

#### 2.2.5. Electron Microscopy

The sections for electron microscopy (50 nm) were obtained using an ultramicrotome (Leica EM UC6 Austria) and were contrasted using a Leica EM AC20 apparatus (Austria) with uranyl acetate and lead citrate solutions (Ultrastain no. 2, Lot 09/138, Laurylab, France). The sections were then analyzed using an electron microscope (Leo 912AB Omega, Germany). 

### 2.3. Immunocytochemical Study

Vibratome sections (10 *μ*m) of brain were fixed with a 4% paraformaldehyde solution in PBS for 2 days. The sections were washed in 0.1% Triton X-100 solution in PBS for 10–15 minutes at room temperature. A ChemiBLOCKER (Lot NMM1737552, Millipore, USA) solution was used for blocking (incubation for 30 minutes). Incubation occurred with primary antibodies against NeuN (mouse antineuronal nuclei, cat. no. MAB377, Millipore, USA) which were diluted at 1 : 500 and maintained for 1 hour at room temperature. Then, the sections were incubated with Image-iT FX signal enhancer (Invitrogen, USA) and were kept in a 1 : 1000 secondary antibody solution with Alexa Fluor 488 goat anti-mouse (from Alexa Fluor 488 Goat-Anti-Mouse SFX kit, lot. 626251, Invitrogen, USA). Incubation with the secondary antibodies lasted for 1 hour in the dark at room temperature. The stained sections were embedded with Fluoromount medium (cat. no. P36930, Sigma). Sections stained according to the same protocol, excluding incubation with primary antibodies, were used as a staining control. The sections were analyzed using a microscope Olympus BX51 (Japan). The MAP2 determination technique was the same as NeuN detection, except for the antibody concentrations. The primary antibody, chicken anti-MAP2 (cat. no. ab5392, Abcam, USA), and the secondary antibody, goat anti-chicken Ig (DyLight 488) (cat. no. ab96951, Abcam, USA), were diluted 1 : 1000. 

### 2.4. Autoradiography

Neurons are characterized by a high transcription rate compared to oligodendrocytes. Thus, the acquisition of neuronal characteristics by an oligodendrocytic nucleus must manifest in the derepression of many genes and an increase in the total rate of RNA synthesis. We tested this hypothesis with an autoradiography experiment. The radioactive RNA precursor uridine-H^3^ was injected into the right ventricle at a dose of 100 *μ*Ci in 20 *μ*L. The precursor was allowed to circulate for 2 hours *in vivo*. 

Sections were obtained using a vibratome and then covered with Amersham EM-1 emulsion (UK). After a 10-day dark exposure, the sections were developed using D-19 developer. The sections were stained with the fluorochrome DAPI, and the resulting epifluorescence allowed determination of the neurons and oligodendrocytes depending on the nucleus structure. To reveal the silver grains, the sample was illuminated by epifluorescence combined with transmitted light. In those combined illumination on a gray background of section, the label (black silver grains) is visible. The precursor spread irregularly in the cortex. Among all of the sections, those in which silver grains were observed only on neurons but not oligodendrocytes were taken for analysis. 

### 2.5. Microgravity Simulation

**Figure 2:**
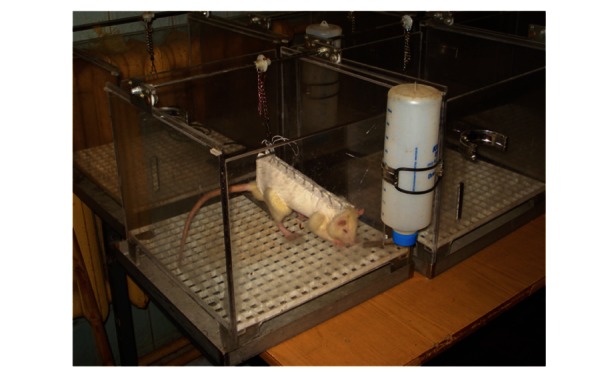
Simulation of microgravitation. Rat from experimental group 4. 1st day of AOH.

Microgravity conditions were simulated by unloading the hind limbs fully and the fore limbs partially by modulation of antiorthostatic hypokinesia (AOH). To simulate microgravity, the rats were equipped with special suits and suspended head down at an angle of 30° in individual cages. The suits were made of synthetic fabric with holes for the fore and hind limbs. A metal plate was stitched into the suit fabric of the dorsal area to provide a straight spine position and to prevent spine wriggle during the experiment. The cage had plexiglass walls and a net floor. The suspension loop was fixed on a block that was freely moved along a joist in the ceiling. This apparatus allowed animals to move easily in the cage and to have full-time access to food and water. Because the hind limbs did not contact the floor, the animals moved using their fore limbs (Figure 2).

### 2.6. Locomotor Activity Analysis

**Figure 3:**
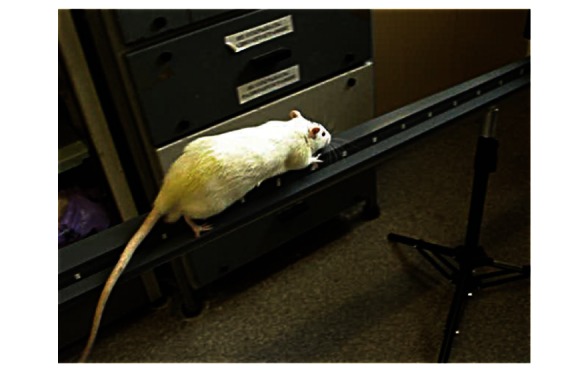
Rat in beam walking locomotor testing system. Rat from experimental group 2. The test of locomotion activity on the 7th day after stroke. Rat forelimbs on the upper plank.

 Locomotor activity was examined before and on the 7th day after the operation, using a “Beam walking" apparatus (OpenScience Ltd, Russia) (Figure 3). 

Installation is composed of a part by which the rat must pass and a dark box closed from all directions with a small entrance that it seeks to reach. The part in which the animal travels represents a combination of two planks, each of 160 cm long. The lower plank is 10 cm wide at the beginning and 5.5 cm at the end. The upper plank is 6 cm wide at the beginning and 1.5 cm at the end. The upper plank is barely attached to the lower plank in such a way that the space from the edge of the lower plank to the edge of the upper plank is a constant width of 2 cm. The box shelter is barely attached to the narrow end of the lower plank. Planks and box are wooden and are painted by safe paint. All of the construction is fixed on two metal tripods at the height of 50 cm from the floor. Experiments with the installation were conducted in the silent spacious room with bright and regular daylight.

Locomotor activity was measured using a semi-quantitative technique with an increased score that reflected exacerbations of locomotor disturbances. The following scale was used.

Passes through the installation in no more than 15 seconds. Accidental slipping of forelimbs—1 point. Such behavior was observed in all control animals and in rats before the experiment.

Systematic slipping of the right forelimb from the upper to lower plank. In the last quarter of the distance, the rat cannot raise a paw on the upper plank—2 points.

Systematic slipping of the right forelimb from the upper to lower plank. In the second half of the distance, the rat cannot raise a paw on the upper plank—3 points.

Movement with a limp. Systematic right forelimb slipping from the upper to lower plank along the whole distance—4 points.

Movement with a strong limp. Do not use the right forelimb. Balance shift to the left part of the body during the motion—5 points.

Right forelimb is disabled; animal loses balance, freezes on the installation, and thrills. Summation of time spent stopped exceeds 6 minutes—6 points.

The animals who have gained less than 6 points always passed installation in less than 6 minutes. 

### 2.7. Experimental Groups

The experiment was conducted using normal rats (control, *n* = 7) and 5 groups of animals that were subjected to the stroke simulation surgery. The animals were distributed into groups with varied injury extents and presence of platelets on transplant. We wanted to investigate neural regeneration for different degrees of damage. The standard damage was achieved by injection of 300 *μ*L of the transplant. In group 1, the transplant contained collagen sponge and acellular plasma from rabbit blood. We hypothesized that the damage would be reduced if platelets containing many angiogenic factors (e.g., VEGF, PDGF, PDAF, TGF-*β*, IGF, FGF, and EGF) were included in the transplant. Therefore, in group 2, the acellular plasma was replaced with PRP. The transplant for the other groups contained collagen sponge and PRP, but the extent of the damage was altered by subjecting animals to AOH. Animals in group 3 received 7 days of AOH after a stroke in which the blood outflow from the head was hampered, resulting in an increased level of damage. Group 4 animals received 7 days of AOH prior to a stroke to verify that the adverse results in group 3 were caused by an influence of AOH on the traumatized vessels. Group 5 animals received 2 days of AOH before a stroke, which served to check the ability of AOH to favorably influence neuronal regeneration. Experimental conditions are presented in Table 1. 

### 2.8. Statistical Analysis

The quantity of di- and heterokaryons was estimated in the semi-thin sections. The section area was then measured using the Cell F software. By dividing the area value by the number of binuclear cells, the mean area (in mm^2^) containing one binuclear cell was determined. The results were compared using the Wilcoxon criterion. The statistical results are reported as the means ± SEM. *P* values lower than 0.01 were considered to be statistically significant. The results of locomotion activity were compared using the Wilcoxon criterion.

## 3. Results

### 3.1. Lethality

 In group 1, three of the nine surgically operated rats died. In group 2, four of the twelve rats died, and in group 3, eighteen of twenty-four died. In group 4, five of twelve rats died, and in group 5, only one rat out of eight died (Table 2).

 The autopsies revealed vast epidural and subdural hemorrhages in the left hemisphere. A morphological analysis of frontal brain sections of the dead rats demonstrated that the hemorrhages spread only to the cerebral cortices and did not penetrate to the white matter or cerebral ventricles. 

The employed hemorrhagic stroke model manifested as a paresis of the right forelimb in all of the animals undergoing an operation. 

An estimation of the locomotor function according to the described score system gave the results that are shown in Table 2. 

### 3.2. Neuropathological Findings

**Figure 4:**
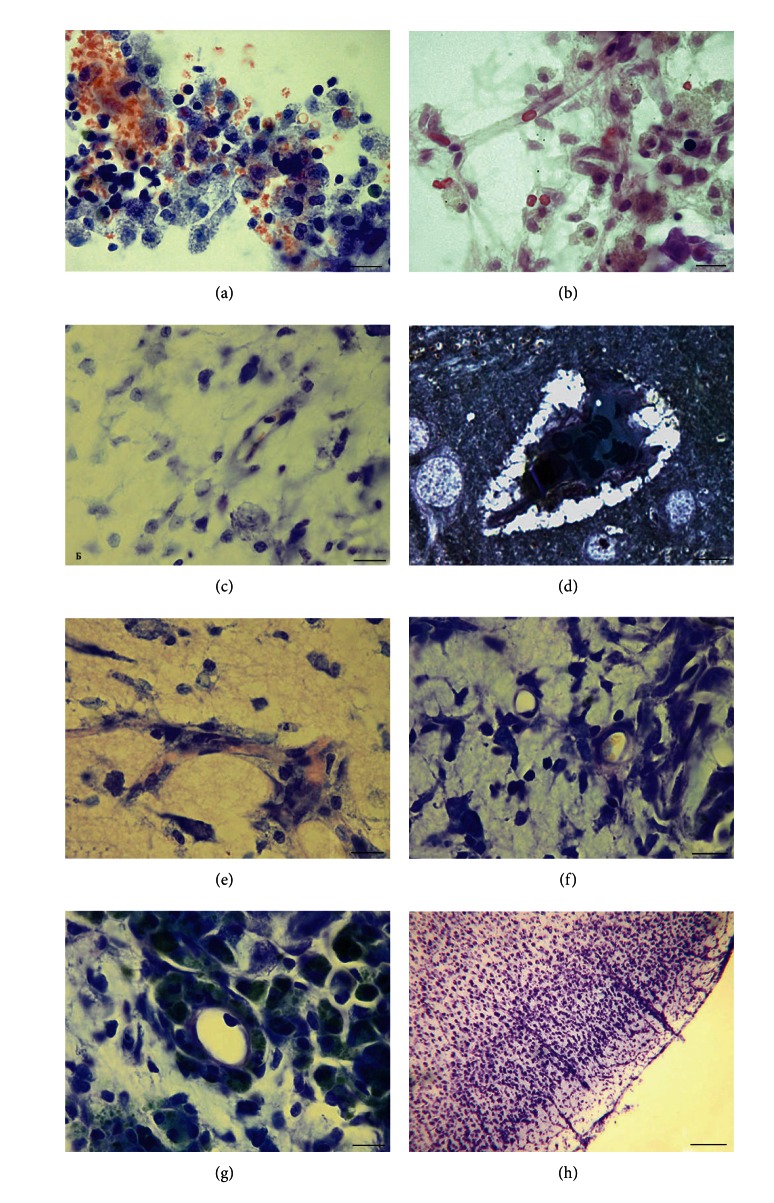
Morphological characteristics of groups 1 and 2. (a) Group 1: an infiltrate zone. Macrophages lying loosely are observed with the cytoplasm containing light granules. Erythrocytes effused out of the vessels in the image. (b) Group 1: infiltrate zone. Macrophages and newly formed capillary tubes are observed with an erythrocyte inside. (c) Group 1: penumbra. The cells of an infiltrate, a neuron, and a vessel with a thick wall in the section. (d) Group 1: penumbra. A blood vessel has hypertrophied walls; a circle of macrophages is absent. Perivascular edema. Semithin section. (e) Group 2: infiltrate zone. Branched vascular tree is filled with erythrocytes. (f) Group 2: penumbra zone. Infiltrate cells and venules with small diameters are observed. The wall of one of the vessels is thickened. (g) Group 2: infiltrate zone. Tightly packed macrophages filled with green granules are observed. Cross-section of a large vessel surrounded by a sphere of macrophages can be identified. (h) Group 2: rxternal part of the cortex, where there are hypertrophied vessels outside of the zone of injury. The scale bars in (a)–(g) are 20 *μ*m, and in (h), 100 *μ*m.

Morphological study of brain tissue from group 1 revealed some signs of circulatory disturbance that continued up to the 7th day after the operation.  Figure 4(a) shows the profuse extravasation, which attracted many macrophages.

Extravasates in the group 1 were observed only in the infiltrate and were not noticed in the penumbra or boundary zone. Not only was vascular tree destruction observed in this group but also its regeneration was apparent, that is, *de novo* vessel growth (Figures 4(b), 4(c), and 4(d)). However, the vessels grew slowly, and in the infiltrate and penumbra; they were rare and had small diameters (capillary tubes, mainly). In the boundary zone, no alterations were found in the vascular tree.

In group 2, the number of vessels in the penumbra was higher than that in group 1 (Figure 4(e)). The vessel walls often looked thickened. The vessels were larger (more mature) and were presented mainly by venules and arterioles as well as by capillary tubes (Figure 4(f)).

Group 2 differed from group 1 in the macrophage reaction. There were a lot of macrophages, and many of them had dark cytoplasm and green-stained granules (metachromasy). Macrophages were often located in the walls of vessels (Figure 4(g)). Vessel hypertrophy in the boundary zone was apparent (Figure 4(h)).

**Figure 5:**
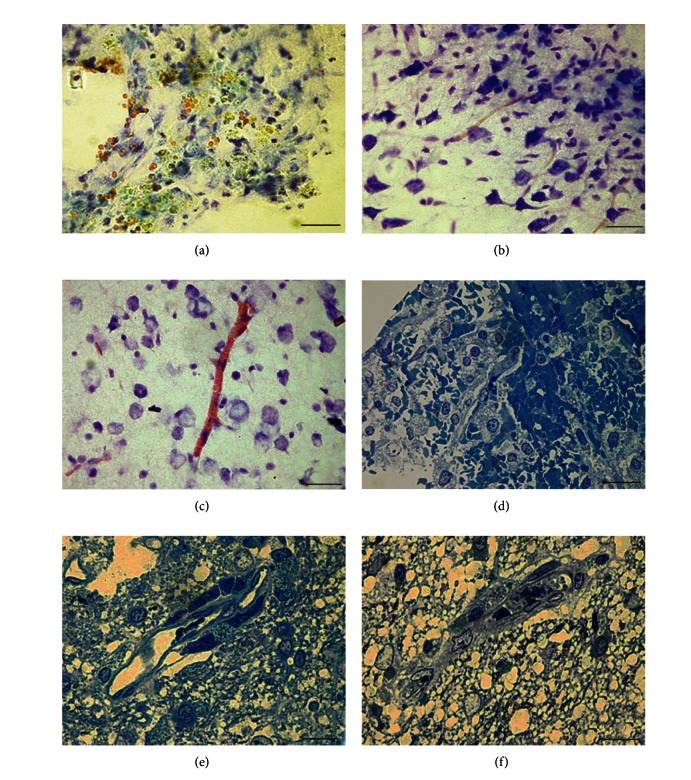
Morphological characteristics of group 3. (a) Group 3: infiltrate zone. Extravascular arrangement of numerous erythrocytes and many macrophages containing green granules. (b) Group 3. boundary zone. Blood stagnation in capillary tubes. (c) Group 3: normal cortex, according to the structure of neurons, surrounding a damaged zone. Noticeably widened capillary tube with erythrocytes accumulated inside and pressed into rouleaux formation. (d) Group 3: extravasate. Macrophages containing pale granules. Semi-thin section. (e) Group 3: blood vessel in the infiltration zone. A circle of macrophages with green granules surrounds the vessel. Semi-thin section. (f) Group 3: blood vessel in the infiltration zone (more destroyed than (e)); a sphere of dark macrophages surrounds the vessel. Semi-thin section. The scale bars in (a)–(f) are 20 *μ*m.

The alterations in the animals of group 3, which were subjected to AOH after the injury, manifested essentially in the morphological characteristics of acute blood flow disturbances. Severe lesion of blood circulation—extravasates which were not observed in group 2—were often found in group 3 (Figures 5(a) and 5(d)). The fact that many longitudinal sections of vessels became clearly distinguished but remained invisible at the normal blood circulation was taken as evidence of outflow disturbances and blood congestion. This phenomenon was observed in the boundary zone as well as in normal cortex surrounding the damage area (Figures 5(b) and 5(c)).

Analysis of the results obtained from group 3 demonstrated that the appearance of a special population of macrophages with green granules is caused by an excess of platelets. Such macrophages were not found in group 1, while in groups with platelets in the center, these macrophages were often numerous (Figure 4(g) and Figures 5(e) and 5(f)). 

One of the main characteristics of groups 2 and 3 is the number of macrophages that contain green granules. In group 2, more macrophages contained green granules. In group 3, macrophages, which were not bounded to the vessel wall, often had pale granules (Figure 5(d)). At the same time, macrophages forming a circle that surrounded the vessels always had green granules (Figures 5(e) and 5(f)). 

**Figure 6:**
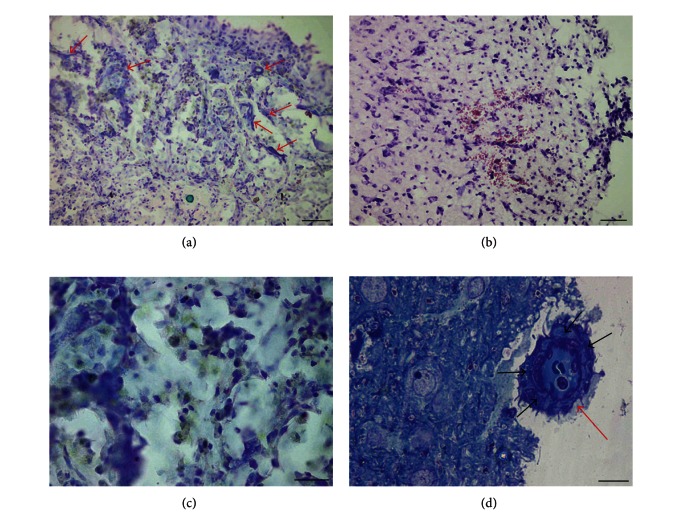
Morphological characteristics of group 4. (a) Group 4: infiltrate. Numerous cells of infiltrate and newly formed vessels. Arterioles are marked with arrows. (b) Group 4: penumbra. Extravasate formed by separated erythrocytes at the boundary between the penumbra and infiltrate. (c) Group 4: infiltrate. Macrophages with pale and stained granules. (d) Group 4: penumbra. Newly formed blood vessel is marked with a red arrow, and macrophages in the vessel wall are marked with black arrows. Hypertrophied vessel wall containing macrophages with dark cytoplasm. Semi-thin section. The scale bars are 100 *μ*m in (a) and (b), 30 *μ*m in (c), and 20 *μ*m in (d).

Group 4 (animals subjected to 7-day AOH before the injury) was characterized by a higher quantity of newly formed vessels compared to groups 1 and 3 (Figure 6(a)). The vessels were represented not only by capillary tubes but also by more mature venules and arterioles (Figures 6(a) and 6(d)).

At the boundary between the infiltration and penumbra in all of the animals, large extravasates were observed. They were made of loosely lying erythrocytes, which rarely formed dense aggregates (Figure 6(b)). The main part of these extravasates was always located in the penumbra. 

Apart from extravasates, no other blood circulation disturbances, such as blood stasis in the capillary tubes or vessels of larger diameter, were noted. The infiltrates contained macrophages with pale and stained granules (Figure 6(c)).

We observed two types of unbound macrophages: those containing pale cytoplasm and unstained granules and those with dark cytoplasm containing green granules. The number of green macrophages was reduced compared to group 2 and was comparable to the content of macrophages in group 3, in which the animals were subjected to posttraumatic AOH.

Macrophages found in the walls of newly formed vessels predominantly contained dark cytoplasm (Figure 6(d)), which is a common feature in groups 2 and 3.

**Figure 7:**
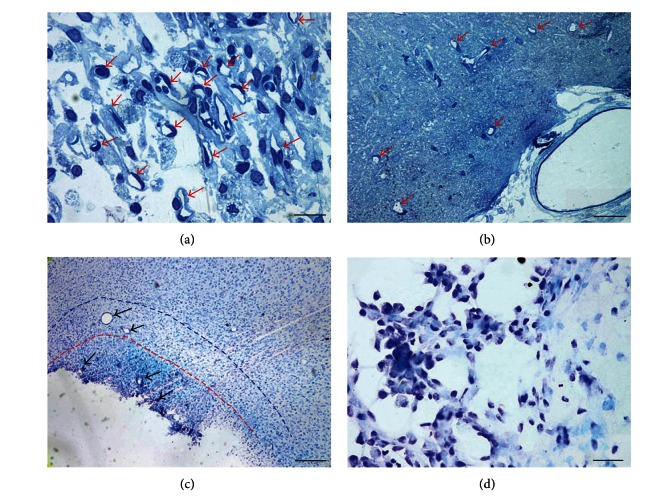
Morphological characteristics of group 5. (a) Group 5. Infiltrate. Newly formed vessels and macrophages. The vessels are marked with arrows. Semi-thin section. (b) Group 5. Penumbra. Newly generated vessels are marked with arrows. Semi-thin section. (c) Group 5. Damaged area. Newly generated vessels with a large diameter are observed in the infiltrate and penumbra. The vessels are marked with black arrows; the red dashed line bounds the infiltration zone, and the blue line denotes the penumbra. (d) Group 5. Infiltration. Small number of macrophages with green granules.  The scale bars are 20 *μ*m in (a) and (d), 100 *μ*m in (b), and 300 *μ*m in (c).

Animals of experimental group 5 had a higher number of newly formed vessels that were found in both the infiltrate and penumbra (Figures 7(a), 7(b), and 7(c)). The infiltrates mainly contained newly formed capillary tubes and sinusoid venules (Figure 7(a)) with extremely thin walls. Arterioles and more mature vessels were found in the penumbra (Figures 7(b) and 7(c)).

In group 5, macrophages with green granules were found only near vessels and in the infiltration, but their number was negligible (Figure 7(d)).

No disturbances of blood circulation in the tissue, such as extravasates or blood congestion, were found. 

In the four experimental groups that received transplants containing platelets, the quantity of cells in the infiltrate can be estimated as being roughly equal.

However, group 1 showed a difference: animals of this group were injected with cell-free rabbit blood plasma. In this group, cells of infiltrate were less than those in the other groups (Figure 4(c)), and the penumbra zone was thin. Another feature of group 1 is an absence of macrophages in the wall of newly formed vessels (Figures 4(c) and 4(d)).

Morphological finds are presented in Table 3.

### 3.3. Binuclear Neurons

 The control animals as well as the experimental animals from all of the studied groups had binuclear neurons in the boundary zone and surrounding cortex. Binuclear neurons of the penumbra were not considered. The occurrence of these neurons in thick (10 *μ*m) sections can be explained by superimposition. However, in semi-thin (1 *μ*m) sections, where superimposing is impossible, cells that contained two identical (dikaryons) or different (heterokaryons) nuclei were found. Nuclei of heterokaryon differed with respect to the size, shape, and chromatin's structure. One nucleus was always a neuronal nucleus, which is large, round, and light. It contained mainly euchromatin and only small granules of heterochromatin. The second nucleus, seen at the sharpest distinction, was small, often spindle-shaped, and dark. It contained rough masses of heterochromatin and was almost deprived of euchromatin. With the highest probability, it is possible to call such a nucleus a nucleus of an oligodendrocyte. The two nuclei in a heterokaryon did not always differ as sharply.

**Figure 8:**
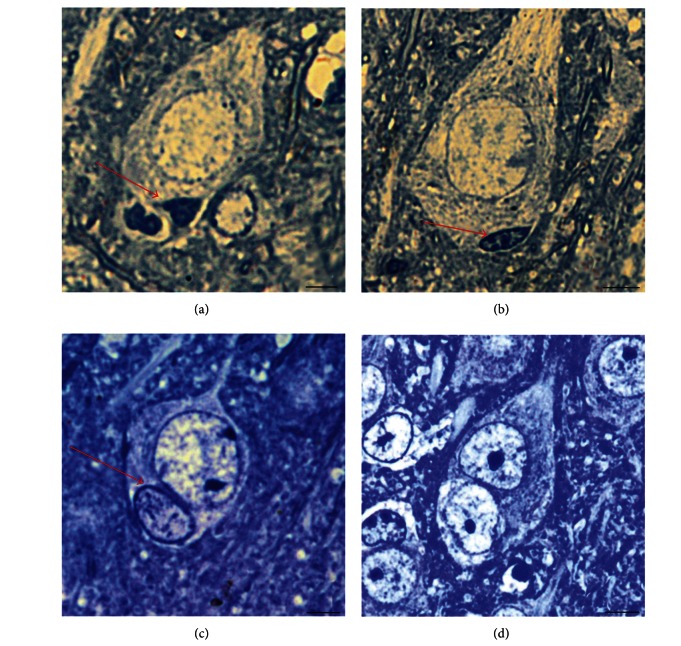
Changes in the structure of the chromatin and the size of the oligodendrocytic nucleus after fusion with a neuron. (a) The oligodendrocytic nucleus is indicated with an arrow. It is located in the neuron's cytoplasm. The nucleus has not changed yet; it is still small, and the chromatin structure cannot be revealed. (b) Beginning of the reprogramming. The size of the nucleus (arrow) did not change, but the chromatin divided into dark heterochromatin and pale euchromatin in the center of the nucleus. (c) The oligodendrocyte's nucleus (arrow) size increased significantly. The heterochromatin belt along the nuclear membrane is wider than that of the neuron's nucleus. (d) Reprogramming completion. The heterokaryon has completely been transformed into a cell that has two identical nuclei—a dikaryon. Semi-thin sections. The scale bar is 10 *μ*m.

Variations in the morphology of oligodendrocytic nuclei can help to identify the individual stages in which they become increasingly similar to neuronal nuclei (Figure 8). 

**Figure 9:**
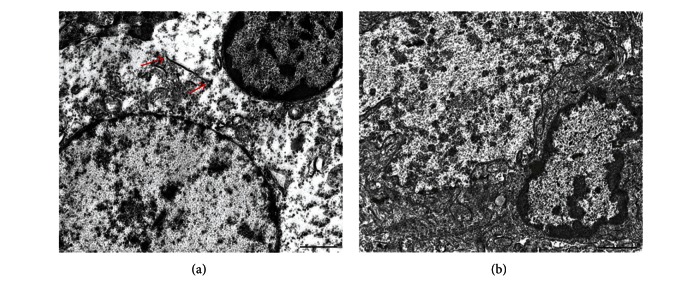
Electron microscope image of heterokaryons. (a) The oligodendrocyte's nucleus (right) and the neuron's nucleus (left) are located in the fused cytoplasm of the two cells. The cell membrane, which previously divided the cells, is observed as a fragment (arrows). The chromatin in the oligodendrocyte's nucleus has divided into heterochromatin (dark accumulations at the nuclear membrane and in the center of the nucleus) and euchromatin (granulated matter at the larger part of the nuclear area). Euchromatin in olygodendrocyte's nucleus darker than euchromatin in neuron's nucleus. (b) The oligodendrocyte's nucleus (right) and the neuron's nucleus (left). Euchromatin in the oligodendrocyte's nucleus has the same light staining as that in the neuron's nucleus. The scale bar is 2 *μ*m.

Electron microscope manifestation of a reprogramming is shown in Figure 9. 

In the cases in which a neuron's nuclear membrane was wrinkled, the oligodendrocyte's nuclear membrane also became wrinkled, which is uncommon for the nuclei of discrete oligodendrocytes (Figure 9(b)).

**Figure 10:**
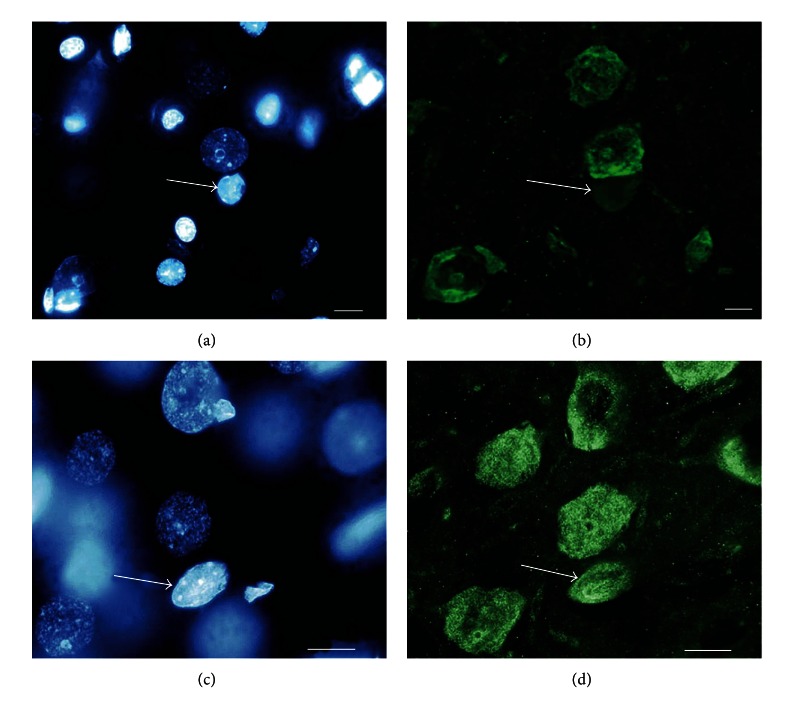
NeuN expression in oligodendrocytic nuclei. (a) In the center of the visual field, a large, dark neuronal nucleus (euchromatin) is observed. A small, bright oligodendrocytic nucleus (heterochromatin) is visible below the nucleus of the neuron (arrow). Ten oligodendrocytes can be observed in the section area, in addition to the nucleus marked with an arrow. (b) The same visual field under a green filter. Neuronal nuclei are green. All 10 extraneous oligodendrocytic nuclei are not visible (they do not contain a neuronal marker), but the oligodendrocytic nucleus under the neuron (arrow) remained visible. It is also immunoreactive to NeuN, but much less so compared to the neuron above. ((c), (d)) A later stage heterokaryon's transformation. In the center of the visible area, a dark, round neuron's nucleus is located near the light, similarly sized oligodendrocytic nucleus (arrow). The large size of the oligodendrocyte's nucleus indicates a long reprogramming duration. (c) The expression level of the neuronal marker NeuN in the oligodendrocyte's nucleus (arrow) does not differ from the neuron's nucleus. ((a), (c)) Staining with the fluorescent dye DAPI. ((b), (d)) Immunocytochemistry with NeuN labeled with Alexa 488. The scale bar is 10 *μ*m.

Oligodendrocytic nuclei not only acquire external similarity with neuronal nuclei but also begin to express specific neuronal markers.  Figure 10 demonstrates the expression of the specific neuronal marker, NeuN protein, in oligodendrocytic nuclei. NeuN immunoreactivity increases along with the degree of structural similarity to neuronal nuclei. 

**Figure 11:**
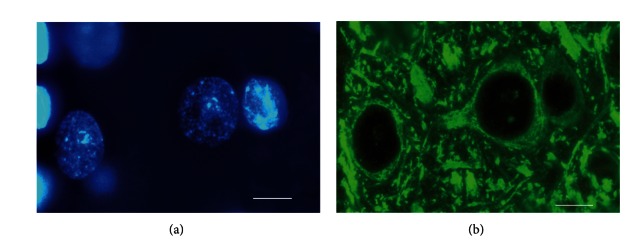
MAP2 expression in a heterokaryon. (a) Neuronal nucleus (larger and darker) and an adjacent reprogramming oligodendrocytic nucleus (smaller in size and brighter). (b) The same section area under a green filter. The circle of green matter (MAP2 immunoreactivity) surrounds the nuclei of both the neuron and the oligodendrocyte. (a) DAPI staining. (b) Immunocytochemical detection of MAP2 labeled by DyLight 488. The scale bar is 10 *μ*m.

We also studied the expression of another neuronal marker, MAP2, in a heterokaryon (Figure 11).

Because MAP2 is a microtubule-associated protein, its immunoreactivity in heterokaryons manifests in accordance with the location of the cell's microtubules, which are revealed as bright-green matter surrounding the neuron's nucleus; microtubules are also apparent in the initial part of the dendrite. A sphere with a less bright-green color of the MAP2-positive matter encircles the nucleus of the oligodendrocyte. 

**Figure 12:**
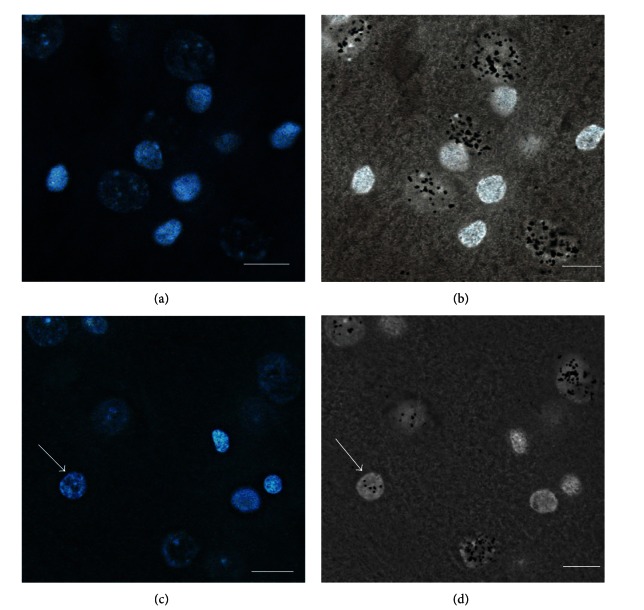
Autoradiography analysis of the RNA synthesis rate in a reprogramming oligodendrocytic nucleus. (a) Here, 5 neuronal nuclei and 6 oligodendrocytic nuclei can be observed in the field of view. (b) The same section area in transmitted light. All 5 neuronal nuclei are intensely marked by silver grains, while none of the 6 oligodendrocytic nuclei contain this label. (c) Another region of the preparation. In the field of view, 4 oligodendrocytic nuclei can be observed. A proneuronal change in the structure, with dark areas of euchromatin, is observed in the nucleus (arrow) that is the largest and is colored irregularly. (d) The same section area in transmitted light. The proneuronal structural change has a functional manifestation: the appearance of the label over the oligodendrocytic nucleus (marked by an arrow) indicates an increase in the RNA synthesis rate. (a) and (c) are stained with DAPI. (b) and (d) show manifestation of the autoradiographic label (silver grains). The scale bar is 15 *μ*m.

The results of autoradiographic experiments are presented in Figure 12, showing an oligodendrocyte's nucleus with a proneuronal alteration in structure (chromatin separation) that changed the function in the proneuronal direction; revealingly, it was labeled as the neuronal nucleus. The transcription rate increased to that of a neuron.

 The transcription of oligodendrocytic genes did not exceed the threshold of perceptibility in our experiment and was not revealed by the label. Therefore, the appearance of a considerable number of silver grains over reprogramming oligodendrocytic nucleus reflects transcription of many neuronal genes within this nucleus.

The proneuronal reprogramming of oligodendrocytic nuclei is a convincing indication that the fusion of regional brain cells plays a role in regeneration. This idea was tested by an analysis of the content of binuclear neurons in the stroke zone during reparative cortex regeneration (Table 2).

Our results suggest that this process is how the regeneration of adult mammalian cortex occurs. Two pieces of evidence support the regenerative value of fusions as follows.

1. The reprogramming of an oligodendrocytic nucleus results in a transformation into the second nucleus of a neuron. Possessing a dual genome increases the functional capacity of the cell and can compensate for age-dependent and pathogenic loss of neurons in the population.
2. The content of binuclear neurons increases in the zone of damage, where favorable conditions for stroke development are formed. The motor activity in this group of animals is quickly restored following the stroke.

## 4. Discussion

 The results presented above show that, during the normal postnatal ontogenesis, regional brain cells, oligodendrocytes, and neurons fuse and form heterokaryons. The oligodendrocytic nucleus in a heterokaryon changes according to the pattern of the neuronal nucleus to which it is fused. The structure of the oligodendrocytic nucleus gradually becomes more similar to that of the neuronal nucleus. The neuronal markers NeuN and MAP2 gradually appear in the oligodendrocytic nucleus and in the surrounding cytoplasm. The transcription rate increases in the oligodendrocytic nucleus to become similar to that of a neuron's nucleus. The observed changes demonstrate the neuron-specific reprogramming of the oligodendrocyte's nucleus. In our opinion, there are sufficient bases to assume that the reprogramming process finishes with the transformation of the initial oligodendrocyte's nucleus into a second neuronal nucleus. The end of reprogramming can be defined by morphological similarity of nuclei in dikaryon. We give one more reason. Two nuclei are in the same cytoplasm under the influence of the same epigenetic regulators. In these conditions full neuron-specific reprogramming of the oligodendrocytic nucleus is inevitable.

Cell fusion in the brain was first described [27–29] on the basis of the results of transgenic experiments, which revealed fusions of donor bone marrow cells (BMDCs) with Purkinje neurons. These publications challenged the notion of postnatal neurogenesis. They proved that the occurrence of a neuron that “transdifferentiated” from a BMDC in the adult brain [30] represents itself an artifact. Transdifferentiation was attributed to the recipient mouse neuron because it contained a transgenic marker—green fluorescent protein (GFP)—that was present in the BMDCs of the donor mouse [30]. The possibility of BMDC fusion proposes another explanation of the experimental results. The BMDCs did not transform into definitive Purkinje cells with the distinguished topography, morphology, and marker expression (glutamate decarboxylase and calbindin) of a differentiated Purkinje neuron. These peculiarities of an “adult” Purkinje neuron were not formed by the BMDC. It simply fused with a differentiated neuron and transferred its GFP marker.

One of the studies of Purkinje neuron fusion [29] showed a reprogramming of BMDCs nuclei in heterokaryons: neuron Purkinje—BMDC. The nucleus undergoing reprogramming grows, the quantity of dispersed chromatin increases, and a Purkinje neuron-specific transgene is activated. The number of heterokaryons increases as a function of the animal's age, which suggests a recovery role [29] for this phenomenon. 

The role of the fusion of Purkinje cells with BMDCs has been discussed as a cellular response to support regeneration, in which an increase in the number of heterokaryons was observed in recipients during chronic inflammation [31].

The phenomenon of regional brain cell fusion that we described differs in many aspects from the fusion of BMDCs with Purkinje neurons. The model of brain regeneration based on the transgenic transplantation results has many weak points.

1. All of the results were obtained in transgenic experiments, which sharply changed the state of the animals via lethal levels of radiation and large doses of immunosuppressors. The conformity of stroke models or other neurologic diseases in such animals is doubtful.
2. Low numbers of fusions were observed (1-2 per 10^6^ of the cells). The highest numbers of fusions were described in the experiment by Johansson et al. [31]; however, hundreds of fusions in the whole cerebellum were observed in only a few animals. So rare events cannot provide reparation.
3. Only bone marrow donor cells take part in the fusions. The role of brain stem cells or any other endogenous cells is uncertain. In transgenic experiments, recipients are irradiated to suppress their stem cells.
4. Irradiation damages the hematoencephalic barrier [32, 33]. Thus, the hypothesis that bone marrow cells penetrated into the brain because of irradiation cannot be rejected; in intact animals, such a penetration is impossible.
5. Fusions involve only Purkinje cells [27–29] and no other types of neurons. Therefore, the regenerative role of fusion was proposed for only one cerebellar neuronal type and not for all of the neurons in the brain.

Here, we revealed a natural process that occurs without extraneous intervention and is independent of enumerated artificial conditions. For identification of the cell that fused with a neuron, we could not use the cytochemical markers that are connected with cytoplasm or the cellular membrane. We couldn't establish the fusing fact until both nucleus won't appear in one cytoplasm and had to determine the second participant of the event simply and solely by the nucleus.

The structure of a nucleus of this participant at an early stage of reprogramming (mainly heterochromatin and lack of a nucleolus) convinces us that it is an oligodendrocyte. Two other cells of a macroglia astrocyte [34, 35] and synantocyte (NG2+ cells) [35–37] have a small amount of heterochromatin that became slightly concentrated under the nuclear envelope and nucleolus. 

The hemorrhagic stroke model that we employed allowed us to examine the regenerative function of binuclear neurons. In groups 3 and 5, the number of binuclear cells in the stroke zone was significantly higher compared to intact animals. Group 5 had a combination of minimal lethality and maximal rate of restoration of the injured limb motion. Morphological analysis helped to identify the positive circulatory changes, that is, a lack of cerebral hemorrhage and blood stasis, in the stroke locus of these animals. The presence of platelets in transplants and 2 days of AOH before the stroke in this group could cause a dense network of newly formed vessels. The frequency of fusions at the injured side was significantly higher compared with the frequency of fusions in the control and in a contralateral hemisphere. An increase in density of binuclear neurons correlated with better functional post-stroke outcome. Locomotor ability almost came back to its initial level before a stroke. A reliable increase in the density of binuclear neurons occurred also in group 3. However, this favorable morphological change was not accompanied, as in group 5, by a fast restoration of function. The damage stimulates regeneration but also blocks it by pathogenic alterations in the locus: hypoxia, excitotoxicity, mechanical stress, and the presence of reactive oxygen species and proinflammatory cytokines, Ca^2+^ overloading [38]. We explain discrepancy of the result for the animals of group 3 with a discrepancy in the action of the damages. The transition of these animals after stroke into AOH increased the afflux and hampered the efflux of blood in the locus [39], enhancing the main pathogenic stroke factor—circulatory distress. As a result, blood disturbance in the zones of the stroke and the lethality of the animals were increased. The animals in groups 4 and 5 were placed into conditions that altered the blood flow prior to the stroke manipulation. The difference between these groups was in the duration of the AOH treatment. AOH within 7 days without the subsequent stroke does not influence significantly the number of binuclear cells [40]. Stroke addition in our experiment did not increase the frequency of fusions in a damage zone after 7 days of AOH (group 4). A decrease in the duration of AOH before a stroke for 2 days was expressed by an increase in the frequency of fusions and a more favorable course of healing in the zone of a stroke (group 5). In animals, group 5 found the maximum number of vessels in the zones of the infiltrate and penumbrae, and there were no hemorrhages. This result, which is in general positive, can presumably be explained by an increase in the intravenous tension under microgravity, which leads to the opening and dilation of collaterals and anastomoses. These alterations appear quickly after gravitation begins and serve to improve the circulation. Longer lasting microgravity stimulates the adverse consequences of the increased venous tension, that is, the reduction in vessel elasticity and edema. Moreover, longer lasting AOH promotes the more significant negative effect of hypokinesia. The positive result observed in group 5 requires further experimental study and could lead to prophylactic and therapeutic developments in the treatment of stroke. 

We describe the natural process of neuron-oligodendrocyte heterokaryon formation. The intrinsic formation of normal cell heterokaryons had not yet been discovered. Changes in an oligodendrocyte's nucleus in a heterokaryon are called neuron-specific reprogramming. Reprogramming is indicated by neuronal changes in the size of a nucleus, the chromatin structure, the appearance in the nucleus and surrounding neuronal markers, and the increased speed of a transcription to be similar to the speed of a neuron. The reprogramming observed in our study is not that of an undifferentiated or stem cell; instead, it is the reprogramming of a definitive, postmitotic oligodendrocyte. The number of fusions observed under the microscope is high enough for quantitative analysis. Certainly, the actual number of fusions is more than what was observed in semi-thin sections because the fusions can be identified only when both nuclei of a dikaryon or heterokaryon lie in the section plane. In the event that a binuclear cell section includes only one nucleus and another nucleus lies lower or higher than the plane of the section, the cell is classified as mononucleate.

We declared, with respect to the completion of reprogramming, the existence of a second nucleus in a cell that was morphologically identical to the first. After the fusion of regional brain cells and the completed reprogramming of the oligodendrocytic nucleus, the second neuron-specific nucleus appears, which is a second genome, in essence. Fusion with oligodendrocytes does not increase the number of neurons in the cortex but instead raises the quantity of the neuronal genomes. Connection between structure and function is a biological expression of connection between matter and movement. Function is a form of existence of structure. They change respectively and synchronously. Autoradiographic experiment confirmed it. Doubling of structure leads to doubling of functions. The neuronal genome dictates neuronal function; thus, with a constant number of neurons, the total functional outcome is amplified because of the presence of some number of binuclear cells. In this way, the death of a subset of neurons during normal ontogenesis or disease can be compensated. Regeneration via fusion preserves the cell body of the neuron and its connections with neighboring neurons. We suppose that nature's strategy in cortical regeneration is aimed not at neuronal digenesis but toward the maintenance of the functional sufficiency of the unique generation.

## 5. Conclusions

Regional cells of the brain, neurons, and oligodendrocytes are often fused with each other in the cortex.

The described mechanism of regeneration not only possesses fundamental meaning but also can be used in practice as a marker in experimental trials for prophylactic, therapeutic, or physiotherapeutic means or in the development of drugs that could have a neurotropic effect. This effect can be quantitatively measured through the state of the regenerative process in the brain by counting binuclear cells. The same technique can also be used to study the effects of pathogenic factors.

## Figures and Tables

**Table 1:** Characteristics of experimental groups.

Experimental groups	Transplant composition	Microgravity action
Control	No	No

1	Collagen sponge + rabbit acellular plasma	No

2	Collagen sponge + rabbit PRP	No

3	Collagen sponge + rabbit PRP	7 days AOH after stroke

4	Collagen sponge + rabbit PRP	7 days AOH before stroke

5	Collagen sponge + rabbit PRP	2 days AOH before stroke

**Table 2:** Results of the quantitative data.

Groups	Number of animals	Lethality, %	Locomotion activity, mean value	Mean area of the section occupied by one binuclear neuron (per mm^2^)	
Control	7	0	1	0.317 ± 0.033	

1	6	33	2.83 ± 0.75	a	0.283 ± 0.0070
				b	0.254 ± 0.0152

2	8	33	1.87 ± 0.64	a	0.250 ± 0.0003
				b	0.234 ± 0.0006

3	6	75	5.33 ± 0.52	a	0.187 ± 0.0002
				b	0.187 ± 0.0013

4	7	42	1.29 ± 0.49	a	0.294 ± 0.0960
				b	0.303 ± 0.0475

5	7	12	1.29 ± 0.49	a	0.195 ± 0.0023
				b	0.181 ± 0.0079
				Contralateral hemisphere	0.315 ± 0.0338

There were significant differences (P < 0.01) between the controls and groups 3 and 5 in the density of binuclear neuron localization. In group 5, the density localization of binuclear neurons in the stroke zone is significantly higher compared to the undamaged hemisphere. Values are reported as the mean ± SEM.

There were significant differences between the locomotion activity in the control group and experimental groups 1, 2 and 3. Groups 4 and 5 did not differ significantly from the control. Group 3 (the maximum average value of the gained points) differed significantly from groups 4 and 5.

**Table 3:** Morphological characteristic groups with stroke.

Groups	Structure of infiltrate	Macrophages in vessel wall	Condition of the vessels	Extravasate
1	Macrophages with light granules only	No	Small number of vessels in infiltrate and penumbra	Small in infiltrate only

2	Macrophages with green granules predominantly	Macrophages with green granules only	Large number of vessels in infiltrate and penumbra; hypertrophied vessels outside the zone of injury	No

3	Macrophages with light and green granules	Macrophages with light and green granules	Large number of vessels in infiltrate and penumbra; blood stagnation in the capillary boundary zone	Extensive in infiltrate and penumbra

4	Macrophages with light and green granules	Macrophages with light and green granules	Large number of vessels in infiltrate and penumbra	Small in penumbra only

5	Macrophages with light granules predominantly	Macrophages with green granules only	Maximal number of vessels in infiltrate and penumbra	No

## Abbreviations

PBS: Phosphate-buffered saline
PRP: Platelet-rich plasma
AOH: Antiorthostatic hypokinesia
VEGF: Vascular endothelial growth factor
PDGF: Platelet-derived growth factor
PDAF: Platelet-derived angiogenic factor
TGF-*β*: Transforming growth factor beta
IGF: Insulin-like growth factor
FGF: Fibroblast growth factor
EGF: Epidermal growth factor
NG2: Neuronalglial antigen 2
BMDC: Bone-marrow-derived cell.
